# A Unique Case of an Aggressive Gangliocytic Paraganglioma of the Filum Terminale

**DOI:** 10.1155/2016/1232594

**Published:** 2016-06-28

**Authors:** Omar S. Akbik, Crina Floruta, Muhammad O. Chohan, Karen S. SantaCruz, Andrew P. Carlson

**Affiliations:** ^1^Department of Neurosurgery, University of New Mexico, Albuquerque, NM 87131, USA; ^2^Department of Pathology, University of New Mexico, Albuquerque, NM 87131, USA

## Abstract

Paragangliomas are rare neuroendocrine tumors that are mostly found in the head and neck. Even less common are gangliocytic variant paragangliomas of the spine for which there are only 7 other documented cases in the literature. We report a case of gangliocytic paraganglioma of the sacral spine in a 68-year-old man. The growth pattern is documented over three years, which to our knowledge has not previously been reported in the literature and is different from the natural history. Clinical, radiological, and pathological characteristics of the tumor are discussed in light of available reports of this rare tumor.

## 1. Introduction

Paragangliomas are rare benign neuroendocrine tumors that can arise from the adrenal medulla or extra-adrenal paraganglia and are characterized histologically by chief cells with an abundance of neurosecretory granules, arranged in lobules surrounded by sustentacular cells [1]. Paragangliomas can arise anywhere in the sympathetic and parasympathetic chain of ganglia with the most common sites in the sympathetic nervous system being para-aortic. Extra-adrenal paragangliomas are also found in the head and neck region (glomus jugulare and carotid body tumors), ampulla of Vater, jejunum, pylorus of stomach, and, rarely, cauda equina [2], where they may account for up to 3.8% of all tumors in that region [3]. In the cauda equina region, this tumor presents as an intradural extramedullary mass. Extra-adrenal paragangliomas may develop a gangliocytic component (gangliocytic paragangliomas), which consist of ganglion cell components in addition to the sustentacular cells [4]. Nearly half of cauda equina paragangliomas contain mature ganglion cells [5]. The origin of this variation remains unclear, although it is generally believed that gangliocytic paragangliomas originate from neuroectodermal ganglion or spindle cells [6]. The diagnosis is confirmed with immunohistochemical staining after surgical resection. Here, we describe a case of a 68-year-old male with an incidental sacral mass that was lost to follow-up and returned with progressively worsening lower extremity paresthesia and a significantly larger lumbosacral mass. To the best of our knowledge this is the first case report to document the growth of a gangliocytic paraganglioma of the spine which is more aggressive than that previously reported in the literature.

## 2. Case Report

### 2.1. Presentation and Examination

**Figure 1:**
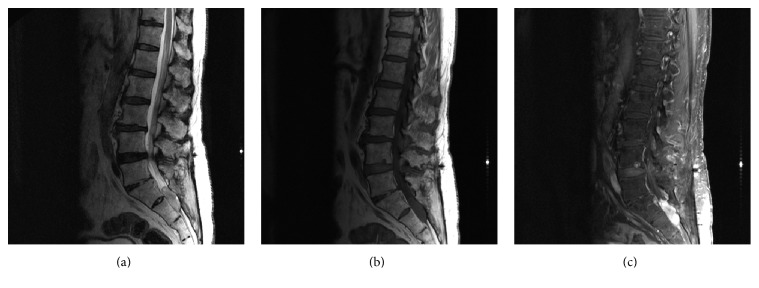
MRI of the lumbar spine with and without contrast. (a) Sagittal T2, (b) sagittal T1, and (c) sagittal T1 after fat saturation: Displays a T1 isointense, T2 hyperintense contrast enhancing mass at the S1-S2 level. The mass measures approx. 3.4 × 1.2 cm without local invasion into surrounding structures.

**Figure 2:**
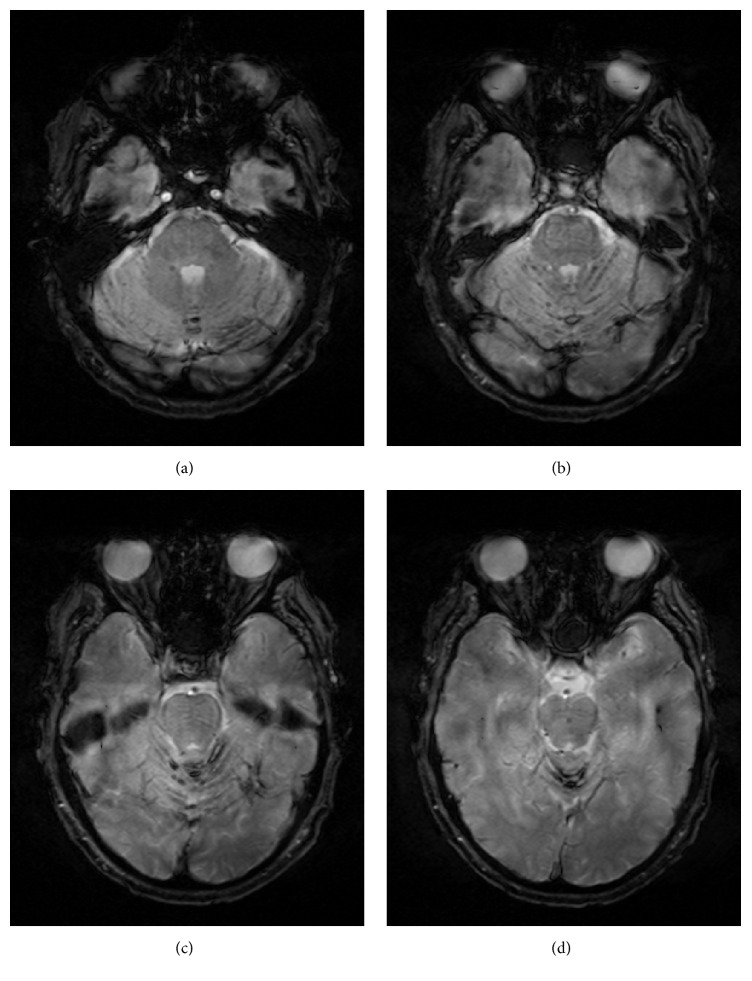
T2-weighted gradient ECHO MRI of the brain: superficial siderosis with hemosiderin deposition seen in the vermis and folia of the cerebellum.

**Figure 3:**
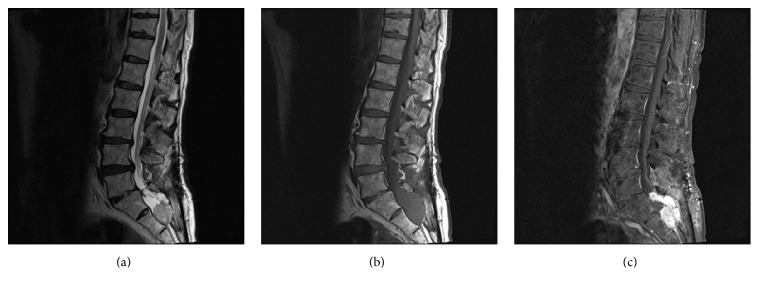
MRI of the lumbar spine with and without contrast. (a) Sagittal T2, (b) sagittal T1, and (c) sagittal T1 after fat saturation: significant enlargement of previously noted sacral mass with new bony erosion and significant mass effect within the spinal canal causing displacement of posterior spinal structures. The mass has taken a more lobulated appearance as seen on the T2 images and is vividly contrast enhancing.

A 68-year-old male with a history of multiple lumbar spine decompressive surgeries for lumbar stenosis and radiculopathy was found to have an incidental sacral mass measuring 3.4 × 1.2 cm located intradurally at the S1, S2 level (Figure 1). An MRI brain showed superficial siderosis primarily in the posterior fossa likely due to hemorrhage from the sacral mass which then deposited in the posterior fossa (Figure 2). A repeat MRI in 6 months was ordered; however, the patient was lost to follow-up until he represented 3 years later to the ED with complaints of perianal paresthesia and significant postvoid residuals. Repeat MRI showed significant enlargement of the sacral mass, which now measured 6.0 × 6.2 cm with an increasingly heterogeneous and lobular pattern. The vividly contrast enhanced lesion now was causing significant compression on the sacral nerve roots (Figure 3).

### 2.2. Operative Findings

The patient underwent L5 through S2 laminectomies for microsurgical resection of the tumor with electrophysiological monitoring. Intraoperatively, it was noted to be beefy and highly vascular in appearance. The lesion was exophytic dorsally from the dura and expanded the sacral nerve root sleeves. The sacral nerve roots were draped both dorsally and ventrally. The filum terminale was identified by its distinct appearance and verified with neurostimulation. The filum entered directly into the mass and was amputated. A gross total resection was achieved and due to the severe disruption of the dura a dural substitute graft was sutured to the muscular edges of the defect in a watertight fashion. Postoperatively, the patient reported marked improvement in his paresthesia in the primarily perianal distribution; however, daily catheterization for urinary retention was required.

### 2.3. Pathological Examination

**Figure 4:**
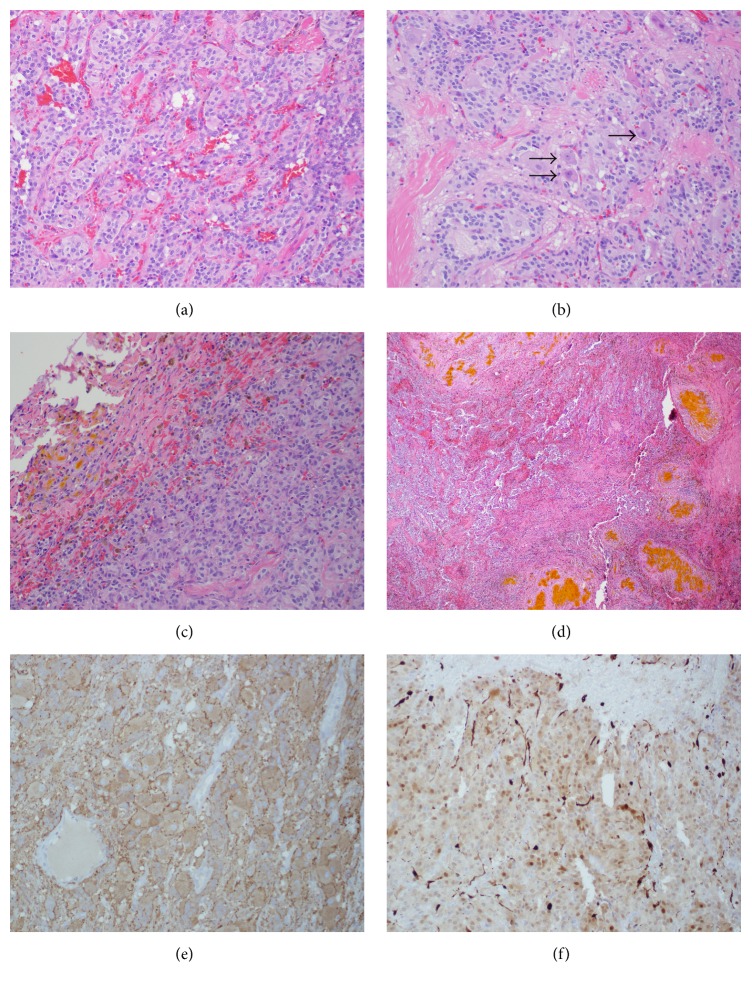
Gangliocytic paraganglioma. (a) Gangliocytic paraganglioma depicting nested arrangement of cells in a Zellballen pattern. H&E stain, 20x. (b) Gangliocytic paraganglioma with ganglion cells (arrow head). H&E stain, 40x. (c) Section of the tumor capsule showing pigmented macrophages (brown) and extracellular hemoglobin breakdown product (yellow). H&E stain, 4x. (d) Focus of remote hemorrhage toward the periphery of the tumor. H&E stain, 20x. (e) Synaptophysin stain positive (brown): positive for synaptic vesicle protein, 20x. (f) S100 stain positive for sustentacular cells (dark brown along edges of lobules), 20x.

Histology revealed islands of uniform tumor cells with neuroendocrine features arranged in a nested Zellballen pattern, which is consistent with a paraganglioma (Figure 4(a)). Additionally, foci of tumor cells with extensive gangliocytic features, including abundant cytoplasm with Nissl-like RNA and prominent nucleoli, were seen (Figure 4(b)). Hemosiderin laden macrophages and extracellular hemoglobin breakdown product were found extensively in the tumor pseudocapsule, with occasional foci of remote hemorrhage within the superficial tumor as well (Figures 4(c) and 4(d)). Immunostaining was negative for glial fibrillary acidic protein (GFAP) and positive for synaptophysin and S100 (Figures 4(e) and 4(f)).

## 3. Discussion

### 3.1. Natural Progression

The first documented description of a paraganglioma in the cauda equina region was in 1972 [12]. There have been several cases of paragangliomas in the cranial and spinal regions since then; however, only 7 cases with gangliocytic variations have been reported, as listed in Table 1. The most frequent presentation is lumbar pain and sciatica with sensory and/or motor deficits along with bladder/bowel dysfunction being less common [13].

Paragangliomas are naturally slow growing tumors, which are most worrisome when located in the head and neck region because of their ability to infiltrate cranial nerves in this area. Paragangliomas in the cauda equina are similar in nature, and these tumors are well-demarcated intradural or extradural masses that do not infiltrate the spinal cord, soft tissues, or bone in the region [11]. This results in an overall good prognosis with gross total resection. The literature reports overall an extremely low recurrence rate with recurrence in some cases occurring after 9 years necessitating prolonged observation due to the slow growing nature of the tumor [1]. It is estimated that 4% will recur following gross total removal [14]. If a subtotal resection is achieved, treatment with radiation is recommended [1].

Jansen et al. estimated the growth rate of head and neck paragangliomas in a comprehensive study and found that a majority of paragangliomas (*n* = 48) had a doubling time of >10 years with an average of 4.2 years [15]. This case differs from the natural history of most paragangliomas in that the growth was more than double during the 3-year interval between studies.

Given the relatively benign natural history, observation may be a reasonable option in some cases of asymptomatic lesions; however, close clinical follow-up is needed in such cases both to rule our more aggressive lesions and due to the potential for growth as in our case.

### 3.2. Radiographic Findings

On MRI, paragangliomas are typically hypo- to isointense on T1-weighted images, hyperintense on T2-weighted images, and vividly enhancing on contrasted studies. Other tumors of the cauda equina including meningioma, schwannoma, and myxopapillary ependymoma can have similar imaging profiles making histologic examination the key to diagnosis. However, due to the hypervascular nature of paragangliomas, a classic “salt and pepper” appearance on T2-weighted images has been described with flow voids interspersed in a matrix of increased signal intensity caused by slow flow and tumor cells [16]. Given enough time, erosion of the adjacent boney structures secondary to chronic bone compression can be seen on plain X-rays or even MRI imaging as shown on Figure 3 on the posterior aspect of the 2nd sacral vertebral body.

Hemorrhage into the tumor has been documented in other case reports either by radiographic or by histologic findings [10, 17]. This case exemplifies not only local hemorrhage within the tumor (Figures 4(c) and 4(d)) but also signs of hemosiderin deposition in the posterior fossa indicating recurrent hemorrhage (Figure 2). Superficial siderosis can be present usually in the posterior fossa, as found in this case, due to deposition of hemosiderin from recurrent hemorrhage. In some cases, this can become symptomatic with sensorineural deficits, cerebellar ataxia, or pyramidal signs [18].

### 3.3. Pathologic Findings

Paragangliomas are comprised of two cell types, chief cells and spindle shaped sustentacular cells, which are classically described as having a “Zellballen” or nesting pattern. In the above case, neurofilament staining in spindle cells is present along with ganglion cells confirming a gangliocytic variation of a paraganglioma. S100 staining is positive in sustentacular cells and can also be positive in tumor cells of ependymomas. GFAP staining can be used to help differentiate the two pathologies because ependymal cells are GFAP positive whereas GFAP staining is negative in neoplastic cells of paragangliomas.

## 4. Conclusion

We report a case of spinal gangliocytic paraganglioma with aggressive growth as compared to the natural history of spinal paragangliomas. Accompanying the radiographic imaging that details growth of this entity, we report the histologic findings which confirm the diagnosis and verify the hemorrhagic nature of this tumor as suspected by the earlier MRI findings of the brain. This case illustrates the uniquely rapid progression of a sacral gangliocytic paraganglioma over a 3-year period.

## Figures and Tables

**Table 1:** Clinical features of reported gangliocytic paragangliomas in cauda equina region.

Case	Age/sex	Clinical presentation	Location	Size (mm)	Pathological findings	Intervention
Current case	68/M	Temporary urinary incontinence, LE, perineal paresthesias	S1-S2 intradural	60 × 26	Neuroendocrine cells in Zellballen pattern, ganglion cells, abundant cytoplasm, GFAP (−), S100 (+), synaptophysin (+)	Complete surgical resection

Vural et al. [3]	17/M	Low back pain, sciatica, difficulty in ambulation	L4 intradural	50 × 30	Neuroendocrine cells in Zellballen pattern, ganglion cells, calcification, GFAP (−), S100 (+)	Complete surgical resection

Llena et al. [7]	42/M	Low back pain, LE weakness	L1 intradural	35 × 20	Neuroendocrine cells in Zellballen pattern, large neurons, neurosecretory granules, dopamine (+)	Complete surgical resection

Matschke et al. [8]	63/F	Low back pain	Cauda equina		Neuroendocrine cells in Zellballen pattern, vascular tissue, ganglion cells, GFAP (+)	Complete surgical resection

Djindjian et al. [9]	36/M	Low back pain, sudden paraplegia following sacral infiltration of medication	L2–L5 intradural	80 × 30	Cells in Zellballen pattern, large mature neurons, gangliocytic differentiation, neurosecretory granules	Complete surgical resection

Mishra et al. [10] described features of 8 paragangliomas in the spinal region, of which two were identified as having prominent gangliocytic differentiation.						

Moran et al. [11] described features of 30 different paragangliomas in the spinal region, of which one is classified as gangliocytic.						

## Additional Points

In this case report, a spinal gangliocytic paraganglioma showed aggressive growth differentiating this variant from the natural history of the filum terminale paraganglioma.

## References

[1] Gelabert-González M. (2005). Paragangliomas of the lumbar region. Report of two cases and review of the literature. *Journal of Neurosurgery: Spine*.

[2] Suresh A. V. S., Varma P. P., Sinha S. (2010). Risk-scoring system for predicting mucositis in patients of head and neck cancer receiving concurrent chemoradiotherapy [rssm-hn]. *Journal of Cancer Research and Therapeutics*.

[3] Vural M., Arslantas A., Isiksoy S., Adapinar B., Atasoy M., Soylemezoglu F. (2008). Gangliocytic paraganglioma of the cauda equina with significant calcification: first description in pediatric age. *Zentralblatt für Neurochirurgie*.

[4] Sirohi D., Sengupta P., Kumar H., Rao P. P. (2010). Gangliocytic paraganglioma: a rare presentation as intestinal intussusception. *Indian Journal of Pathology & Microbiology*.

[5] Burger P. C., Scheithauer B. W. (1994). Tumors of paraganglionic tissue: tumors of the central nervous system. *Atlas of Tumor Pathology*.

[6] Nakamura T., Ozawa T., Kitagawa M. (2002). Endoscopic resection of gangliocytic paraganglioma of the minor duodenal papilla: case report and review. *Gastrointestinal Endoscopy*.

[7] Llena J. F., Wisoff H. S., Hirano A. (1982). Gangliocytic paraganglioma in cauda equina region, with biochemical and neuropathological studies. Case report. *Journal of Neurosurgery*.

[8] Matschke J., Westphal M., Lamszus K. (2005). November 2004: intradural mass of the cauda equina in a woman in her early 60s. *Brain Pathology*.

[9] Djindjian M., Ayache P., Brugieres P., Malapert D., Baudrimont M., Poirier J. (1990). Giant gangliocytic paraganglioma of the filum terminale. Case report. *Journal of Neurosurgery*.

[10] Mishra T., Goel N. A., Goel A. H. (2014). Primary paraganglioma of the spine: a clinicopathological study of eight cases. *Journal of Craniovertebral Junction and Spine*.

[11] Moran C. A., Rush W., Mena H. (1997). Primary spinal paragangliomas: a clinicopathological and immunohistochemical study of 30 cases. *Histopathology*.

[12] Lerman R. I., Kaplan E. S., Daman L. (1972). Ganglioneuroma-paraganglioma of the intradural filum terminale. Case report. *Journal of Neurosurgery*.

[13] Scheithauer B. W., Brandner S., Soffer D. (2007). Spinal paraganglioma. *WHO Classification of Tumours of the Central Nervous System*.

[14] Strommer K. N., Brandner S., Sarioglu A. C., Sure U., Yonekawa Y. (1995). Symptomatic cerebellar metastasis and late local recurrence of a cauda equina paraganglioma: case report. *Journal of Neurosurgery*.

[15] Jansen J. C., Van Den Berg R., Kuiper A., Van Der Mey A. G. L., Zwinderman A. H., Cornelisse C. J. (2000). Estimation of growth rate in patients with head and neck paragangliomas influences the treatment proposal. *Cancer*.

[16] Olsen W. L., Dillon W. P., Kelly W. M., Norman D., Brant-Zawadzki M., Newton T. H. (1987). MR imaging of paragangliomas. *American Journal of Roentgenology*.

[17] Yang S.-Y., Jin Y. J., Park S. H., Jahng T. A., Kim H. J., Chung C. K. (2005). Paragangliomas in the cauda equina region: clinicopathoradiologic findings in four cases. *Journal of Neuro-Oncology*.

[18] Fearnley J. M., Stevens J. M., Rudge P. (1995). Superficial siderosis of the central nervous system. *Brain: A Journal of Neurology*.

